# Maternal CMV seroprevalence rate in early gestation and congenital cytomegalovirus infection in a Chinese population

**DOI:** 10.1080/22221751.2021.1969290

**Published:** 2021-09-14

**Authors:** Yue Huang, Tingdong Li, Huan Yu, Jiabao Tang, Qiaoqiao Song, Xiaoyi Guo, Han Wang, Caihong Li, Jiangding Wang, Caihong Liang, Xingmei Yao, Lingxian Qiu, Chunlan Zhuang, Zhaofeng Bi, Yingying Su, Ting Wu, Shengxiang Ge, Jun Zhang

**Affiliations:** a The State Key Laboratory of Molecular Vaccinology and Molecular Diagnostics, National Institute of Diagnostics and Vaccine Development in Infectious Diseases, Collaborative Innovation Center of Biologic Products, School of Public Health, Xiamen University, Xiamen, People’s Republic of China; b Xinmi Maternal and Child Health Hospital, Xinmi, People’s Republic of China; c Jiaxian Maternal and Child Health Hospital, Jiaxian, People’s Republic of China; d Zhongmu Maternal and Child Health Hospital, Zhongmu, People’s Republic of China

**Keywords:** Cytomegalovirus, congenital infection, prevalence, seroprevalence, pregnant women

## Abstract

Background Congenital human cytomegalovirus (CMV) infection remains largely unrecognized and underemphasized in medical practice. This study aimed to describe the maternal CMV seroprevalence rate in early gestation and congenital CMV infection in a Chinese population. Methods This prospective cohort study was conducted in three hospitals in China from 2015 through 2018. Pregnant women were enrolled in early gestation and followed up in middle and late gestation with serological testing. CMV serostatus was determined by IgG testing in serum during early gestation. Their newborns were screened for cCMV infection by PCR testing in both saliva and urine at two time points. The cCMV prevalence, maternal seroprevalence and associated factors were analyzed. Results In China, the CMV seroprevalence was 98.11% (6602/6729, 95% CI: 97.76%–98.41%), and the cCMV prevalence was 1.32% (84/6350, 95% CI: 1.07%–1.64%). Over 98% of cCMV-positive newborns were from pregnant women who were seropositive in early gestation in China. The prevalence of cCMV infection in newborns from seropositive and seronegative pregnant women was similar (crude prevalence: 1.33% vs 0.82%, *P* = 1.00; estimated prevalence: 1.27% vs 1.05%, *P* = 0.32). Pregnant women who were under 25 years old or primiparous had a lower seroprevalence. Newborns from pregnant women under 25 years old or from twin pregnancies had a higher prevalence of cCMV infection. Conclusion in China, the cCMV prevalence was high, and the rates were similar in newborns from pregnant women who were seropositive and seronegative in early gestation. The vast majority of cCMV newborns were from seropositive mothers.

**Trial registration:**ClinicalTrials.gov identifier: NCT02645396..

## Introduction

Congenital human cytomegalovirus (cCMV) infection remains largely unrecognized and underemphasized in medical practice, although it is a major infectious cause of sensorineural hearing loss and neurodevelopmental abnormalities in infants. Screening for cCMV infection in newborns is rarely performed in developing countries. There is a consensus that the diagnosis of cCMV infection should be based on positive results at two points in time by the analysis of samples (either saliva or urine) collected within 21 days of life, given the possibility of a false-positive result from the first test [1]. However, the confirmatory testing of subsequent samples is not commonly performed according to previously conducted screening reports, and no such studies have been performed in China. Although, several studies conducted in China have aimed to understand the prevalence of cCMV infection, the unstandardized methods in studies involving the testing of a single kind of sample (dried blood spots, saliva or urine) or detection at a single time point have led to large variation in the reported prevalence, ranging from 0.23% to 6.13% [2–4]. Hence, the true prevalence of cCMV infection in China is still unclear.

While both primary CMV infection in seronegative pregnant women and nonprimary CMV infection in seropositive pregnant women may give rise to vertical transmission, the transmission rates are different. Maternal primary infection will lead to a higher rate of vertical transmission than nonprimary infection, but the incidence of primary infection in seronegative pregnant women is lower than that of nonprimary infection in seropositive pregnant women [5]. However, there has never been a direct comparison of the cCMV prevalence between seronegative and seropositive pregnant women in China, even though this knowledge would be of great significance when considering preventive measures. Moreover, although nonprimary infection in seropositive mothers causes the vast majority of cCMV cases globally, the current measures to protect against cCMV infection are focused mostly on seronegative pregnant women.

To gain a better understanding and take adaptive measures to prevent cCMV infection, this study aimed to demonstrate the attribution of cCMV infection to seropositive versus seronegative pregnant women. From 2015 to 2018, we conducted a cohort study to evaluate the epidemiology of cCMV infection in China and compared the prevalence of cCMV infection in newborns from seropositive and seronegative pregnant women as a reference for regions with extremely high CMV seroprevalence.

## Materials and methods

### Subject recruitment and follow-up

A multicenter prospective cohort study was conducted in three hospitals in Xinmi, Jiaxian and Zhongmu counties in Henan Province, China, from June 2015 to May 2018. Pregnant women attending their first pregnancy check were approached for enrolment. Enrolled women were followed through delivery. Maternal serum specimens were collected at enrolment and in middle and late gestation for CMV-IgG serological testing. Saliva and urine specimens were collected from newborns within 13 days of birth and tested by real-time PCR for CMV DNA. Additional saliva and urine samples typically within 21 days of birth were collected if the first CMV DNA screening test showed a positive result in urine and/or saliva for subsequent confirmatory testing.

The study was approved by the Ethics Committee of the School of Public Health, Xiamen University (ClinicalTrials.gov, NCT02645396). Informed consent was obtained from each participant at the time of enrolment.

### Definition of congenital CMV infection

CCMV infection was confirmed as positive on both screening within 13 days of birth and confirmatory testing within 21 days of birth. Newborns who were not followed up after testing positive on screening within 13 days of birth were defined as suspected of having cCMV infection. Newborns who were positive on screening within 13 days of birth and showed a positive result in confirmatory testing that was conducted beyond 21 days of birth were defined as being highly suspected of having cCMV infection. Newborns who were negative in both saliva and urine on screening or positive in urine and/or saliva on screening but negative in both saliva and urine on confirmatory testing were deemed to not have cCMV infection.

### Cytomegalovirus serology in pregnant women

Serum samples were tested for IgG antibody against CMV by a well-validated enzyme-linked immunosorbent assay, as previously reported [6]. The CMV serostatus of pregnant women was defined by IgG testing in the serum during early gestation. Pregnant women with positive CMV-IgG at early gestation were defined as CMV seropositive; pregnant women with negative CMV-IgG at early gestation were defined as CMV seronegative.

### Cytomegalovirus DNA detection in newborn saliva and urine samples

Saliva specimens from newborns were collected at least one hour after feeding by swabbing the inside of the mouth using a sterile cotton swab, softly and sufficiently, until soaked with saliva. The saliva swabs were placed in transport medium (DMEM) immediately after collection. Urine specimens were collected using infant urine drainage bags (GuanKe Bio, NingBo, China), and fecal contamination was carefully avoided.

To prevent DNA degradation, 8 mM ethylenediaminetetraacetic acid (EDTA) was added to the saliva or urine samples, and DNA was extracted from these samples using a Total Nucleic Acid Isolation Kit (GenMag, Beijing, China) according to the manufacturer’s instructions. Then, CMV DNA was quantitated by real-time PCR using one of three pairs of primers and probes targeting UL123 and UL54, respectively, as previous reported [7].

### Statistical analysis

The prevalence of cCMV infection and its 95% confidence limits were the mainly parameters analyzed in this study. The crude prevalence was calculated, where the numerator included the confirmed cCMV cases and highly suspected cCMV cases and the denominator was newborns who underwent cCMV screening by both saliva and urine sampling.

**Figure 1.**
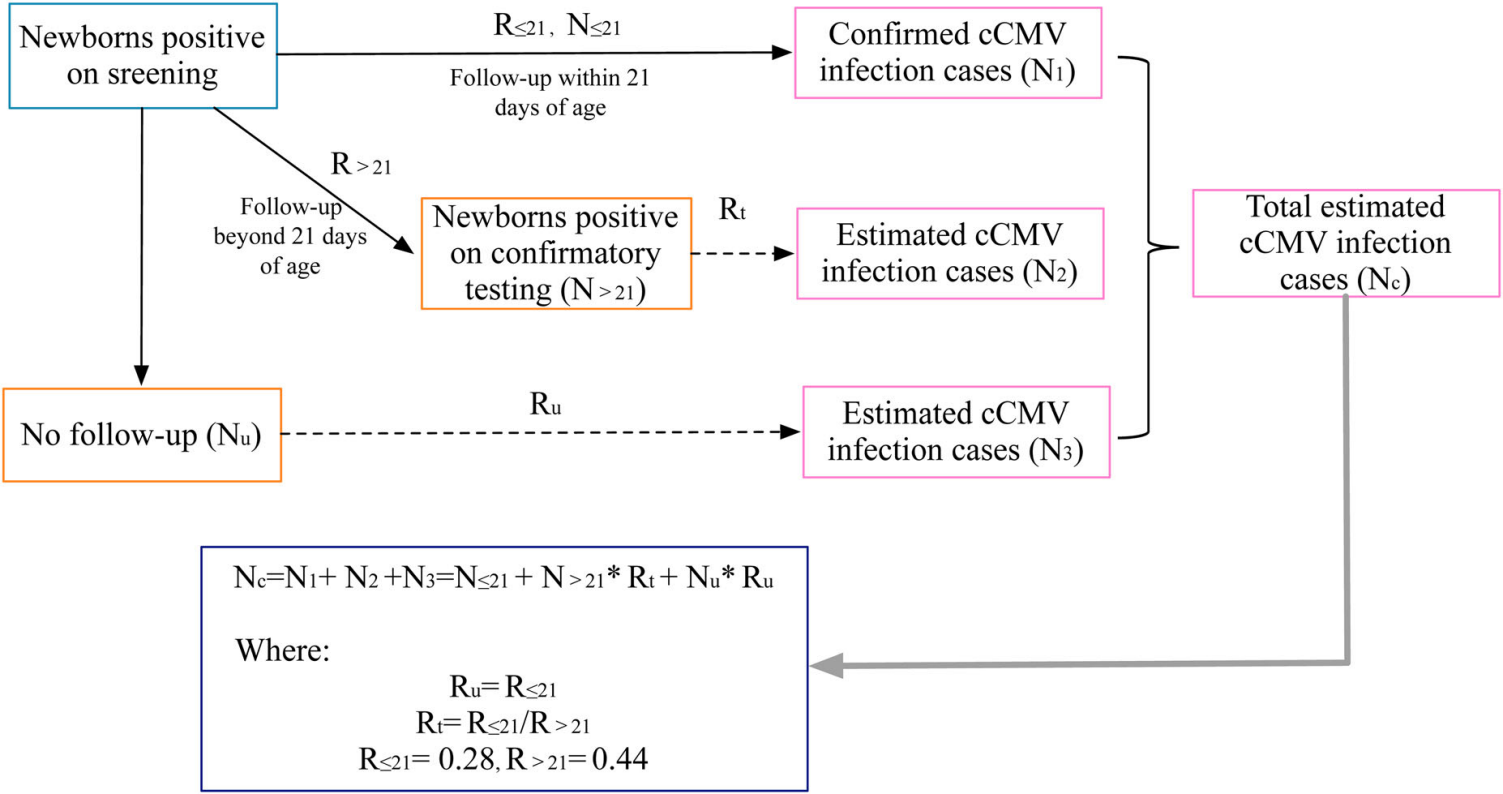
Estimation of the prevalence of cCMV. Note: N_u_ is the number of newborns not followed up after showing a positive result on screening, and R_u_ is the rate of cCMV infection in newborns without follow-up. N_≤21_ is the number of newborns who tested positive on confirmatory testing within 21 days of birth, and R_≤21_ is the rate of cCMV cases in newborns who underwent confirmatory testing within 21 days of birth. N_>21_ is the number of newborns who tested positive on confirmatory testing beyond 21 days of birth, and R_>21_ is the rate of cCMV cases in newborns who underwent confirmatory testing beyond 21 days of birth. R_t_ is the rate of true cCMV cases in newborns who tested positive beyond 21 days of birth on confirmatory testing.

Furthermore, an estimated prevalence was calculated. The estimated number of cCMV cases among the 6350 newborns who underwent cCMV screening was calculated by means of the formula showed in Figure 1. It was estimated that the percentage of cCMV cases among newborns who were positive on screening but not followed up with by confirmatory testing or who were followed up after 21 days of birth would be similar to that among newborns undergoing confirmatory testing within 21 days of birth, since similar demographic characteristics were observed (eTable 1, Supplemental material). Hence, the estimated cCMV cases included the following: ① cases confirmed within 21 days of birth; ② true cases among highly suspected cases (newborns positive on screening within 13 days of birth and on confirmatory testing but beyond 21 days of birth), with a rate of R_≤21_/R_>21_, as there were numerous false-positive cases due to postnatal or perinatal CMV infection; and ③ true cases among suspected cases (newborns positive on screening within 21 days of birth but who were not followed up), where the rate of cases was considered equal to R_≤21_.

Pearson’s chi-square tests or Fisher’s exact tests were used to determine differences in the distribution of categorical variables. In the analysis of sociodemographic factors related to maternal CMV seroprevalence or cCMV prevalence, multiple logistic regression analysis was conducted if more than one variable was statistically associated. *P*-values < 0.05 were considered to indicate statistical significance. All statistical analyses were performed using SAS software (version 9.4; SAS Institute, Cary, NC).

## Results

### Study cohort

**Figure 2.**
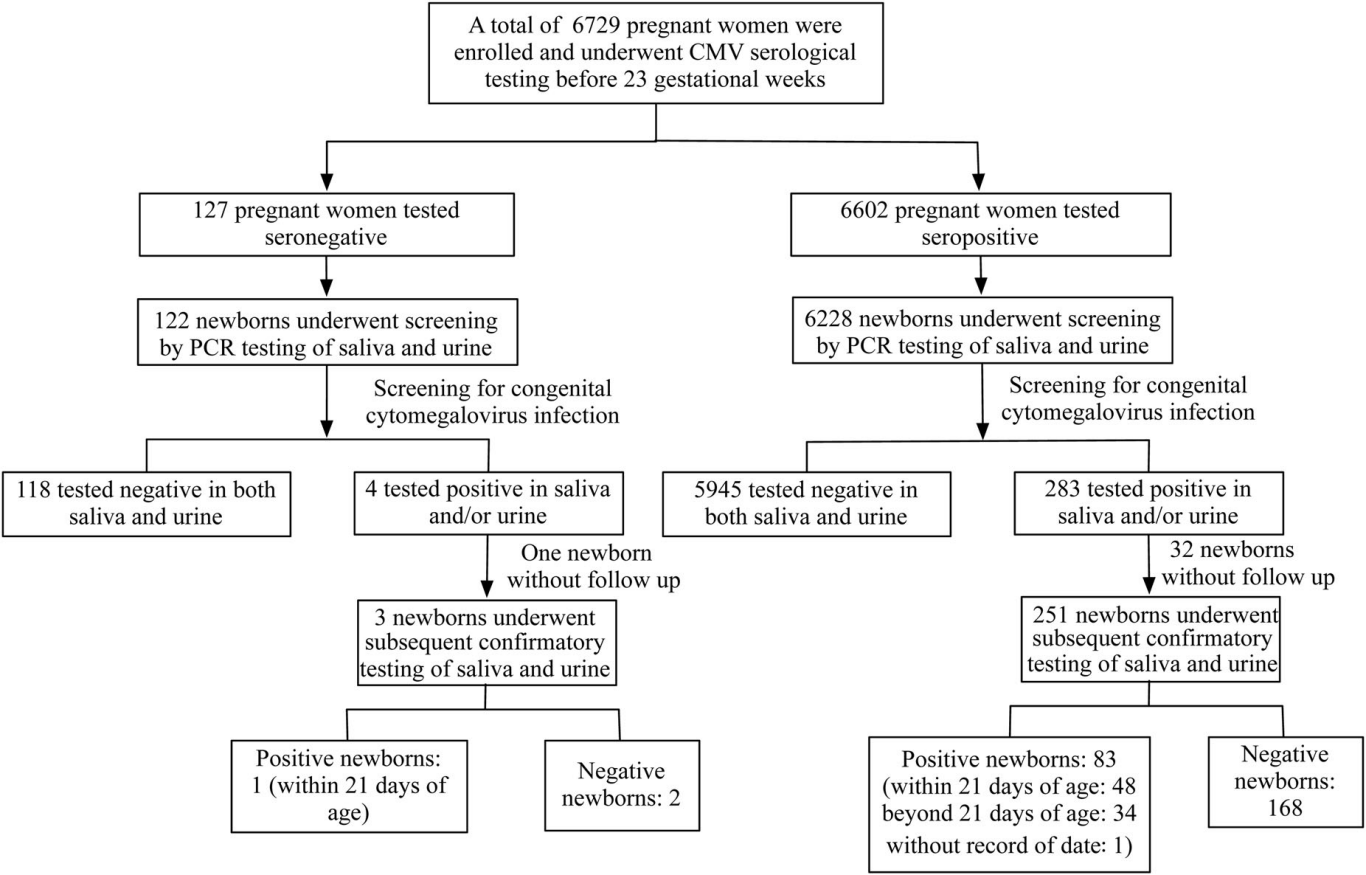
Flow diagram of enrolment and cCMV screening.

A total of 6729 pregnant women underwent CMV serological testing in early gestation (Range of gestational weeks [gw]: 6.0–25.4; median gw: 13.6). Among them, 127 were born to seronegative pregnant women, and 6602 were born to seropositive pregnant women; 6350 newborns underwent cCMV screening with both saliva and urine samples within 13 days of birth (Figure 2).

### Prevalence of congenital cytomegalovirus infection

Among 6350 newborns who underwent cCMV screening, 49, 33 and 35 newborns were confirmed cCMV infection cases, suspected cCMV cases, and highly suspected cases, respectively. The crude prevalence of cCMV infection was 1.32% (84/6350, 95% CI: 1.07%–1.64%) in Henan Province, China, which was the percentage of confirmed and highly suspected cCMV cases. The estimated prevalence of cCMV infection was 1.27% (80.51/6350, 95% CI: 1.02%–1.57%) by the method shown in Figure 1.

Among newborns from seropositive pregnant women, the percentages of cCMV-positive, highly suspected cCMV-positive, suspected cCMV-positive and cCMV-negative newborns were not significantly different from the distribution of newborns from seronegative pregnant women, as shown in Table 1. The crude and estimated prevalence in newborns from seropositive pregnant women was 1.33% (83/6228, 95% CI: 1.08%–1.65%) and 1.27% (79.23/6228, 95% CI: 1.02%–1.58%), respectively, which was not significantly different from that among newborns from seronegative pregnant women (crude prevalence: 0.82%, 1/122, 95% CI: 0.14%–4.50; estimated prevalence: 1.05%, 1.28/122, 95% CI: 0.22%–4.88%). Almost all cCMV-positive newborns were from seropositive pregnant women (crude rate: 98.81%, 83/84, 95% CI: 93.56%–99.79%; estimated rate: 98.41%, 79.23/80.51, 95% CI: 92.74%–99.67%).

**Table 1.** CCMV by maternal serostatus in early pregnancy

	Maternal IgG status		
	Seropositive *n* (%)	Seronegative *n* (%)	*P*-value
Number	6228 (98.1)	122 (1.9)	-
Newborn cCMV status			
Positive	48 (0.77)	1 (0.82)	0.82
Highly suspected positive	35 (0.56)	0 (0.00)	
Suspected positive	32 (0.51)	1 (0.82)	
Negative	6113 (98.16)	120 (98.36)	

Note: Newborns who were not followed up after testing positive in saliva and/or urine on screening were defined as being suspected of having cCMV. Newborns who were positive on screening but who underwent confirmatory testing beyond 21 days of birth were defined as being highly suspected of having cCMV. The samples for confirmation of highly suspected cases were collected from 22 to 51 days of birth with a median age of 27 days, including one newborn whose confirmatory samples were not recorded on the date of collection.

Among the 110 seronegative pregnant women, 6 had CMV seroconversion during pregnancy (5.45%, 6/110, 95% CI: 2.52–11.39), and among them, there was one case of vertical transmission to the newborn (16.67%, 1/6, 95% CI: 3.01–56.35). Two of the 6 women had seroconversion in the first half of pregnancy, two had seroconversion in the second half of pregnancy, and two had an unclear seroconversion time as lacking serological testing in the middle pregnancy (including the case of vertical transmission of CMV).

### Sociodemographic factors relevant to maternal CMV seroprevalence

Among the 6729 pregnant women who were enrolled and underwent CMV serological testing, 6602 were seropositive, with a seroprevalence of 98.11% (95% CI: 97.76, 98.41). Pregnant women under 25 years old had a lower seroprevalence (97.28%) than those over 25 years old (98.39%) (Table 2). The seroprevalence in primiparous pregnant women was significantly lower than that in multiparous pregnant women (97.74% vs 98.76%). The above differences were still significant in multiple logistic regression analysis, with *P*-values of 0.016 and 0.032 for maternal age and parity, respectively.

**Table 2.** Sociodemographic factors relevant to maternal CMV seroprevalence.

	CMV-IgG serostatus (*N*=6729)			
	Positive, *n* (%)	Negative, *n* (%)	*P*-value^1^	*P*-value^2^
Overall	6602 (98.11)	127 (1.89)	–	–
Maternal age in years*				
<25	1788 (97.28)	50 (2.72)	0.003	0.016
≥25	4701 (98.39)	77 (1.61)		
Residence				
Rural area	3900 (98.09)	76 (1.91)	0.90	–
Urban area	2679 (98.13)	51 (1.87)		
First pregnancy*				
Yes	2604 (97.89)	56 (2.11)	0.25	–
No	3132 (98.31)	54 (1.69)		
First live birth*				
Yes	3676 (97.74)	85 (2.26)	0.006	0.032
No	2065 (98.76)	26 (1.24)		

Note: *Missing values: maternal age, 113; residence, 23; first pregnancy, 883; first live birth, 877. ^1^The *P*-value was calculated by means of Pearson’s chi-square tests or Fisher’s exact tests. ^2^The *P*-value was calculated by means of multiple regression analysis.

### Sociodemographic factors relevant to congenital CMV infection

The crude and estimated prevalence of cCMV infection in groups with different demographic characteristics was analyzed, as shown in Table 3. Newborns from mothers under 25 years of age had a significantly higher prevalence of cCMV infection than newborns from mothers over 25 years of age. Additionally, the prevalence of cCMV infection in singletons was significantly lower than that in twins.

**Table 3.** Sociodemographic factors relevant to congenital cytomegalovirus infection

	In total	Confirmed or highly suspected cCMV cases	Crude prevalence of cCMV infection (%)	Estimated prevalence of cCMV infection (%)	P_1_#	P_2_#
Maternal age in years*						
<25	1747	36	2.06	1.89	0.002	0.006
≥25	4570	48	1.05	1.03		
Residence*						
Rural area	3730	49	1.31	1.26	0.87	0.90
Urban area	2569	35	1.36	1.29		
Maternal first pregnancy*						
Yes	2645	43	1.63	1.59	0.28	0.15
No	3187	41	1.29	1.15		
Maternal first live birth *						
Yes	3738	60	1.61	1.54	0.15	0.08
No	2100	24	1.14	0.99		
Preterm birth (<37 weeks) *						
Yes	201	5	2.49	2.25	0.19	0.21
No	6086	79	1.30	1.24		
Sex*						
Male	2967	43	1.45	1.38	0.96	0.8
Female	2862	41	1.43	1.31		
Perinatal asphyxia*						
No	5763	84	1.46	1.36	–	–
Yes	44	0	0.00	0.00		
Weight at birth in grams*						
<2500	162	5	3.09	2.57	0.2	0.38
2500–4000	5174	72	1.39	1.30		
≥4000	493	7	1.42	1.43		
Singleton pregnancy*						
Yes	6226	78	1.25	1.22	0.001	0.002
No (twins)	91	6	6.52	4.85		

Note: *Missing values: maternal age, 33; residence, 51; first pregnancy, 518; first live birth, 512; preterm birth, 63; type of delivery, 512; sex, 521; perinatal asphyxia, 543; weight at birth, 521; singleton pregnancy, 33. # P_1_: test for difference in crude prevalence of cCMV among different groups for each variable; P_2_: test for difference in estimated prevalence of cCMV among different groups for each variable.

## Discussion

This was a large-scale multicenter cohort study in which a strict design was applied to understand the epidemiology of cCMV infection in China. Both saliva and urine samples were collected for CMV detection in newborns, and subsequent confirmatory testing was conducted to exclude false-positive results. Hence, in this study, a relatively accurate estimated prevalence of cCMV infection in China, namely, 1.27% (80.51/6350, 95% CI: 1.02%–1.57%), was calculated. In addition, the distribution of cCMV infection among newborns from seropositive and seronegative pregnant women was determined. The vast majority (98.81%) of cCMV-positive newborns were from seropositive pregnant women, while the prevalence of cCMV infection was similar in newborns from seropositive and seronegative pregnant women.

The prevalence of cCMV infection was higher in newborns from mothers under 25 years old (1.89%) and higher in twins (4.85%), which is consistent with findings from other studies [3,8]. Other sociodemographic factors related to cCMV infection that have been reported include race, multipartner sexual activity, fewer previous pregnancies, crowded living environments and frequent exposure to children [3,8–11]. Since it has been reported that the cCMV prevalence in newborns is positively correlated with the seroprevalence in mothers [12], we also analyzed the CMV seroprevalence in populations with different sociodemographic characteristics. Pregnant women who were under 25 years old or primiparous had a lower seroprevalence, although it was still extremely high (over 97%). In terms of maternal age, the lower seroprevalence in mothers under 25 years old might be related to the higher prevalence of cCMV infection. The antibody level at early gestation was lower in seropositive women < 25 years old than in those ≥25 years old (10.6 IU/mL vs 12.4 IU/mL, *P*<0.001). It is speculated that the higher frequency of reinfection in women under 25 years due to the higher viral exposure via an active lifestyle and a lower protective immunity, such as lower seroprevalence and a lower antibody level, might together give rise to the higher cCMV prevalence in mothers younger than 25 years old.

The reported prevalence of cCMV infection in China varies among different studies (0.23% to 6.13%) [2–4]. Differences in laboratory methods, type of samples analyzed, and criteria for cCMV confirmation may account for the variations. Detection has been applied for a single kind of sample (dried blood spots, saliva or urine) or at a single time point in most screening programmes. Saliva and urine are suggested samples for cCMV screening. Although saliva and urine supplement each other in recognizing cCMV cases, the difficulty of urine collection in newborns limits its application in screening programmes. Confirmatory testing of subsequent samples collected within 21 days of birth following a positive screening result to exclude false-positive results was suggested by the International Congenital Cytomegalovirus Recommendations Group [1]. In this study, a comprehensive and strict strategy was used to identify cCMV-positive newborns by analyzing both saliva and urine samples collected within 21 days of birth for screening and confirmatory testing. The prevalence reported in this study is similar to that found in Brazil by a well-done population-based screening study (1.08%, 87/8047) [13] in which saliva and/or urine samples were collected for the first analysis and again for confirmatory testing. The prevalence of cCMV infection is related to CMV seroprevalence in populations; therefore, the prevalence of cCMV infection in China and Brazil is similar, as the similar seroprevalences (both over 95%) [14].

Maternal primary infection and nonprimary infection led to different rates of vertical transmission. The dogma in which cCMV-positive infants born to seropositive mothers develop less adverse clinical outcomes has led to the neglect of cCMV-affected infants in the seropositive population. However, two meta-analyses demonstrated that the risk of hearing loss in cases of maternal nonprimary infection and maternal primary infection was approximately 11% and 13%, respectively, and the predictive model showed that the worldwide contribution of nonprimary infections in causing CMV-related hearing loss was greater than that of primary infections [12,15]. According to the China Statistical Yearbook, the number of newborns each year is approximately 15 million, and it is estimated that cCMV-positive newborns account for approximately 190,000 cases. Thus, approximately 187,000 cCMV-positive newborns may be born to seropositive pregnant women, while approximately 3000 may be born to seronegative pregnant women. Without any preventive measures, 21.0 thousand newborns may develop hearing loss of different severities.

Worldwide prevention measures, including research into a potential vaccine, have focused mainly on seronegative pregnant women. In China, preventive measures for cCMV infection are lacking. Accurate awareness of cCMV infection is missing among most pregnant women and even among medical staff; additionally, cCMV screening is rarely performed, which perpetuates the lack of awareness of cCMV infection and related conditions. Studies are needed to highlight these issues, and adaptive measures should be considered. The similar prevalence of cCMV infection in newborns from seropositive and seronegative pregnant women found in this study truly reflects the similar risk of vertical transmission in both populations, and measures for seropositive pregnant women should also be considered, as the vast majority of cCMV-positive newborns were from seropositive pregnant women. There is still a long way to go to achieve sufficient understanding and prevention of cCMV infection, and a series of scientific questions need to be answered. The disease burden needs to be demonstrated more clearly in regions with high CMV seroprevalence, and predictive indicators of the risk of vertical transmission in the seropositive population need to be explored. Other factors that need further exploration include the role of maternal reinfection and reactivation in cCMV infection and related clinical outcomes, the efficacy of potential CMV vaccines for seropositive pregnant women in the prevention of cCMV infection, and the cost-effectiveness of preventive strategies.

There are several limitations to this study. Due to the major challenge of following up with all newborns with a positive screening result within 21 days of birth, some newborns with a positive screening result were not followed up at all or within 21 days of birth. The specific estimation was performed to compensate for missing or biased data and to produce an accurate estimation of the prevalence as much as possible. In addition, anti-CMV testing to define a history of exposure to CMV in pregnant women was conducted in the early phase of pregnancy instead of before pregnancy, and some participants were beyond 12 weeks at the time of enrolment. Hence, a small proportion of seronegative pregnant women infected with CMV who underwent seroconversion in very early gestation might have been included in the seropositive population; however, this would barely affect the final results, as the primary infection rate is low within a short period of time. (One study reported a seroconversion rate of 0.26% (5/1951) between 12 and 36 gw [16]). Additionally, the seroprevalence is extremely high among child-bearing females in China [17,18]. Notably, the population of women who were seronegative in early gestation was small, which led to reduced precision in the estimation of the cCMV prevalence. Last, the initially positive screening results of some infants were not confirmed, and the reasons for this are unclear; however, potential contamination was excluded after analyzing the data related to processes of sample collection and detection [7]. Of these newborns, the majority had a low viral load in saliva or urine. The nonnegligible false-positive result in screening is an issue also reported in other studies [19–22], which is the reason to recommend confirmatory testing using subsequent samples in the current consensus [1,23]. Herein, we confirmed the newborns’ cCMV status according to the consensus’s suggestion; however the long-term clinical outcomes of these questionable newborns will be followed up in the future to better understand their cCMV status.

In summary, this study demonstrates the epidemiology of cCMV infection in newborns in China and the attribution of cCMV infection to seropositive and seronegative pregnant women, which is vital information for considering preventive strategies.

## Supplementary Material

Supplemental_materials.docx

## References

[1] Rawlinson WD, Boppana SB, Fowler KB, et al. Congenital cytomegalovirus infection in pregnancy and the neonate: consensus recommendations for prevention, diagnosis, and therapy. Lancet Infect Dis. 2017;17(6):e177–e188. 28291720 10.1016/S1473-3099(17)30143-3

[2] Zhang XW, Li F, Yu XW, et al. Physical and intellectual development in children with asymptomatic congenital cytomegalovirus infection: a longitudinal cohort study in Qinba mountain area, China. J Clin Virol. 2007;40(3):180–185. 17919973 10.1016/j.jcv.2007.08.018

[3] Wang S, Wang T, Zhang W, et al. Cohort study on maternal cytomegalovirus seroprevalence and prevalence and clinical manifestations of congenital infection in China. Medicine (Baltimore). 2017;96(5):e6007. 28151899 10.1097/MD.0000000000006007 PMC5293462

[4] Collaborative Team for the Study of Maternal and Infantile Cytomegalovirus Infection in Beijing. Prospective study on the impact of infantile cytomegalovirus infection on growth and development of infants. Chinese Journal of Pediatrics. 2010;48(5):385–389. 20654046

[5] Huang Y, Song Q, Guo X, et al. Risk factors associated with the vertical transmission of cytomegalovirus in seropositive pregnant women. Future Virol. 2019;14(4):265–273.

[6] Huang X, Li JJ, Ge SX, et al. Establishment and validation of an enzyme-linked immunosorbent assay for IgG antibody against cytomegalovirus based on pp150 antigen. J Virol Methods. 2017;240:21–25. 27825854 10.1016/j.jviromet.2016.11.001

[7] Huang Y, Wang H, Li T, et al. Comparison of detection strategies for screening and confirming congenital cytomegalovirus infection in newborns in a highly seroprevalent population: a mother-child cohort study. Lancet Reg Health West Pac. 2021:12:100182. 10.1016/j.lanwpc.2021.100182 PMC8356112 34527973

[8] Leruez-Ville M, Magny JF, Couderc S, et al. Risk factors for congenital cytomegalovirus infection following primary and nonprimary maternal infection: a prospective neonatal screening study using polymerase chain reaction in Saliva. Clin Infect Dis. 2017;65(3):398–404. 28419213 10.1093/cid/cix337

[9] Kharrazi M, Hyde T, Young S, et al. Use of screening dried blood spots for estimation of prevalence, risk factors, and birth outcomes of congenital cytomegalovirus infection. J Pediatr. 2010;157(2):191–197. 20400091 10.1016/j.jpeds.2010.03.002

[10] Fowler KB, Pass RF. Risk factors for congenital cytomegalovirus infection in the offspring of young women: exposure to young children and recent onset of sexual activity. Pediatrics. 2006;118(2):e286–e292. 16847076 10.1542/peds.2005-1142

[11] Dorfman JR, Balla SR, Pathirana J, et al. In utero human cytomegalovirus infection is associated with increased levels of putatively protective maternal antibodies in nonprimary infection: evidence for boosting but not protection. Clin Infect Dis. 2021;73(4):e981–e987. 33560335 10.1093/cid/ciab099

[12] de Vries JJ, van Zwet EW, Dekker FW, et al. The apparent paradox of maternal seropositivity as a risk factor for congenital cytomegalovirus infection: a population-based prediction model. Rev Med Virol. 2013;23(4):241–249. 23559569 10.1002/rmv.1744

[13] Mussi-Pinhata MM, Yamamoto AY, Moura Brito RM, et al. Birth prevalence and natural history of congenital cytomegalovirus infection in a highly seroimmune population. Clin Infect Dis. 2009;49(4):522–528. 19583520 10.1086/600882 PMC2778219

[14] Yamamoto AY, Castellucci RA, Aragon DC, et al. Early high CMV seroprevalence in pregnant women from a population with a high rate of congenital infection. Epidemiol Infect. 2013;141(10):2187–2191. 23200458 10.1017/S0950268812002695 PMC9151393

[15] Goderis J, De Leenheer E, Smets K, et al. Hearing loss and congenital CMV infection: a systematic review. Pediatrics. 2014;134(5):972–982. 25349318 10.1542/peds.2014-1173

[16] Delforge ML, Costa E, Brancart F, et al. Presence of cytomegalovirus in urine and blood of pregnant women with primary infection might be associated with fetal infection. J Clin Virol. 2017;90:14–17. 28319846 10.1016/j.jcv.2017.03.004

[17] Huang Y, Guo X, Song Q, et al. Cytomegalovirus shedding in healthy seropositive female college students: A 6-month longitudinal study. J Infect Dis. 2018;217(7):1069–1073. 29294037 10.1093/infdis/jix679

[18] Li TD, Li JJ, Huang X, et al. Baseline antibody level may help predict the risk of active human cytomegalovirus infection in a HCMV seropositive population. Eur J Clin Microbiol Infect Dis. 2016;36(5):863–868. 28032284 10.1007/s10096-016-2873-8

[19] Blázquez-Gamero D, Soriano-Ramos M, Vicente M, et al. Prevalence and clinical manifestations of congenital cytomegalovirus infection in a screening program in Madrid (PICCSA study). Pediatr Infect Dis J. 2020;39(11):1050–1056. 32773658 10.1097/INF.0000000000002808

[20] Eventov-Friedman S, Manor H, Bar-Oz B, et al. Saliva real-time polymerase chain reaction for targeted screening of congenital cytomegalovirus infection. J Infect Dis. 2019;220(11):1790–1796. 31310307 10.1093/infdis/jiz373

[21] Putri ND, Wiyatno A, Dhenni R, et al. Birth prevalence and characteristics of congenital cytomegalovirus infection in an urban birth cohort, Jakarta, Indonesia. Int J Infect Dis. 2019;86:31–39. 31207385 10.1016/j.ijid.2019.06.009

[22] Nagel A, Dimitrakopoulou E, Teig N, et al. Characterization of a universal screening approach for congenital CMV infection based on a highly-sensitive, quantitative, multiplex real-time PCR assay. PLoS One. 2020;15(1):e0227143. 31917817 10.1371/journal.pone.0227143 PMC6952102

[23] Pesch MH, Kuboushek K, McKee MM, et al. Congenital cytomegalovirus infection. Br Med J. 2021;373:n1212. 34083180 10.1136/bmj.n1212

