# Supplementary material for: Maternal CMV seroprevalence rate in early gestation and congenital cytomegalovirus infection in a Chinese population
Source: Emerg Microbes Infect. 2021 Sep 14;10(1):1824–31. doi: 10.1080/22221751.2021.1969290 (PMC8451685; doi:10.1080/22221751.2021.1969290)
Supplement: Supplemental_materials.docx [file TEMI_A_1969290_SM3600.docx]

**Supplemental materials**

**eTable 1. Demographic characteristics of newborns with different definitions of cCMV infection**

|  | Newborns | | | | P_1_^#^ | P_2_^#^ |
| --- | --- | --- | --- | --- | --- | --- |
|  | In total | Confirmed cases  n (%) | Suspected cases  n (%) | Highly suspected cases  n (%) |  |  |
| Maternal age in years | |  |  |  |  |  |
| <25 | 47 | 20 (40.82) | 16 (45.71) | 11 (33.33) | 0.65 | 0.49 |
| ≥25 | 70 | 29 (59.18) | 19 (54.29) | 22 (66.67) |  |  |
| Residence |  |  |  |  |  |  |
| Rural area | 67 | 30 (61.22) | 19 (54.29) | 18 (54.55) | 0.52 | 0.54 |
| Urban area | 50 | 19 (38.78) | 16 (45.71) | 15 (45.45) |  |  |
| Maternal first pregnancy | |  |  |  |  |  |
| Yes | 61 | 27 (55.1) | 16 (45.71) | 18 (66.67) | 0.40 | 0.33 |
| No | 50 | 22 (44.9) | 19 (54.29) | 9 (33.33) |  |  |
| Maternal first live birth | |  |  |  |  |  |
| Yes | 92 | 38 (77.55) | 33 (71.74) | 21 (77.78) | 0.14 | 0.98 |
| No | 30 | 11 (22.45) | 13 (28.26) | 6 (22.22) |  |  |
| Preterm birth (<37 weeks) | |  |  |  |  |  |
| Yes | 6 | 3 (6.12) | 2 (5.71) | 1 (3.03) | 1.00 | 0.66 |
| No | 111 | 46 (93.88) | 33 (94.29) | 32 (96.97) |  |  |
| Sex |  |  |  |  |  |  |
| Male | 59 | 26 (53.06) | 17 (48.57) | 16 (59.26) | 0.68 | 0.60 |
| Female | 52 | 23 (46.94) | 18 (51.43) | 11 (40.74) |  |  |
| Perinatal asphyxia | |  |  |  |  |  |
| No | 111 | 49 (100) | 35 (100) | 27 (100.00 ) | - | - |
| Yes | 0 | 0 (0) | 0 (0) | 0 (0) |  |  |
| Weight at birth in grams | |  |  |  |  |  |
| <2500 | 6 | 2 (4.08) | 3 (8.57) | 1 (3.7) | 0.83 | 1.00 |
| 2500-4000 | 95 | 42 (85.71) | 30 (85.71) | 23 (85.19) |  |  |
| ≥4000 | 10 | 5 (10.2) | 2 (5.71) | 3 (11.11) |  |  |
| Singleton pregnancy | |  |  |  |  |  |
| Yes | 110 | 48 (97.96) | 30 (85.71) | 32 (96.97) | 0.08 | 1.00 |
| No (twins) | 7 | 1 (2.04) | 5 (14.29) | 1 (3.03) |  |  |

Note: # P_1_: P-value of the comparison of the distribution of each demographic factor between groups of confirmed cases and highly suspected cases; P_2_: P-value of the comparison of the distribution of each demographic factor between groups of confirmed cases and suspected cases.
